# Distinct Strains of *Toxoplasma gondii* Feature Divergent Transcriptomes Regardless of Developmental Stage

**DOI:** 10.1371/journal.pone.0111297

**Published:** 2014-11-13

**Authors:** Matthew McKnight Croken, Yanfen Ma, Lye Meng Markillie, Ronald C. Taylor, Galya Orr, Louis M. Weiss, Kami Kim

**Affiliations:** 1 Department of Microbiology and Immunology, Albert Einstein College of Medicine, Bronx, New York, United States of America; 2 Department of Pathology, Albert Einstein College of Medicine, Bronx, New York, United States of America; 3 Environmental Molecular Sciences Laboratory, Pacific Northwest National Laboratory, Richland, Washington, United States of America; 4 Computational Biology and Bioinformatics Group, Biological Sciences Division, Pacific Northwest National Laboratory, Richland, Washington, United States of America; 5 Department of Medicine, Albert Einstein College of Medicine, Bronx, New York, United States of America; University of Wisconsin Medical School, United States of America

## Abstract

Using high through-put RNA sequencing, we assayed the transcriptomes of three different strains of *Toxoplasma gondii* representing three common genotypes under both *in vitro* tachyzoite and *in vitro* bradyzoite-inducing alkaline stress culture conditions. Strikingly, the differences in transcriptional profiles between the strains, RH, PLK, and CTG, is much greater than differences between tachyzoites and alkaline stressed *in vitro* bradyzoites. With an FDR of 10%, we identified 241 genes differentially expressed between CTG tachyzoites and *in vitro* bradyzoites, including 5 putative AP2 transcription factors. We also observed a close association between cell cycle regulated genes and differentiation. By Gene Set Enrichment Analysis (GSEA), there are a number of KEGG pathways associated with the *in vitro* bradyzoite transcriptomes of PLK and CTG, including pyrimidine metabolism and DNA replication. These functions are likely associated with cell-cycle arrest. When comparing mRNA levels between strains, we identified 1,526 genes that were differentially expressed regardless of culture-condition as well as 846 differentially expressed only in bradyzoites and 542 differentially expressed only in tachyzoites between at least two strains. Using GSEA, we identified that ribosomal proteins were expressed at significantly higher levels in the CTG strain than in either the RH or PLK strains. This association holds true regardless of life cycle stage.

## Introduction

*Toxoplasma gondii* is an obligate intracellular parasite belonging to the phylum Apicomplexa. It has a complicated life cycle marked by sexual reproduction in the gastrointestinal tract of a feline host and asexual replication in any warm-blooded animal [1]. The asexual cycle itself is divided into a fast-growing tachyzoite stage and slow-growing bradyzoite stage. The bradyzoite form is thought to persist indefinitely within infected hosts within cysts and can reactivate if a host's immune function wanes. The ability to shift between acute and chronic phases of its life cycle is critical for disease pathogenesis and thus the subject of intense investigation [1].

One mark of the success of *Toxoplasma gondii* is its global distribution. It is estimated to infect around 30% of the human population. The parasite is transmitted through water contaminated with cat feces as well as being clonally propagated from animal to animal via carnivorism. The transmission strategies of *T. gondii* has led to a complex structure of populations within the species. Fourteen different haplogroups have been identified around the world with each lineage containing multiple distinct strains [2]. Research has focused on parasites belonging to groups 1, 2, and 3 (also designated types I, II, and III) isolated in North America and Europe. Although these parasites diverged relatively recently (∼10 kya) [33], they are marked by distinct differences in phenotype, most prominently virulence. Type I tachyzoites are less able to convert to bradyzoites, thereby causing acute disease in their hosts [34]. Type III strains readily differentiate causing their hosts to become chronically infected, but these strains are infrequently associated with clinical disease in humans [34]. Type II strains tend to be intermediate to types I and III in terms of differentiation competence and virulence [34]. Despite significant differences in phenotypes, there appears to be very little difference in genome sequence [3]. Forward genetics studies have attributed most of the difference in virulence to the ROP18 and ROP5 loci [4] [5] [6].

Few studies currently exist describing the differences among the transcriptomes of *T. gondii* lineages and how these transcriptomes change following differentiation from tachyzoite to bradyzoite [25]. What studies do exist are often stymied by lack of gene annotation. Roughly half of *T. gondii* genes are described only as “hypothetical proteins”. Gene Set Enrichment Analysis (GSEA) is a software tool designed to test whether functionally related sets of genes are collectively up or down regulated between experimental conditions [7].

It has been shown that *T. gondii*, like other Apicomplexa, exerts tight control over gene expression [8], but little is known about the effectors that make this control possible. In 2005, a family of genes containing the AP2 DNA-binding domain were identified in the phylum Apicomplexa [9]. Follow up work found 68 AP2 genes in *T. gondii* [10]. Members of this family regulate important life cycle developments in *Plasmodium* [11] [12] [13] [14] and *Toxoplasma* [15]. As many more of these genes are expected to be key transcription factors, observed differences in AP2 expression levels between strains and across life cycle development are of keen importance.

## Results and Discussion

### Inter-strain differences in gene expression much larger than those between tachyzoites and bradyzoites

**Figure 1:**
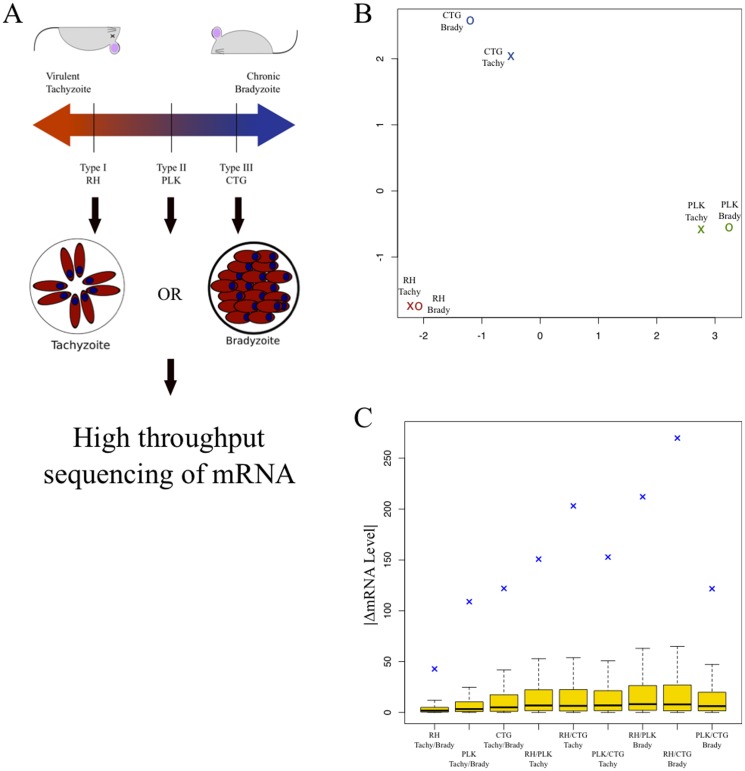
Patterns of gene expression vary more by strain than by developmental stage. A) A representative strain from *T. gondii* lineages Types I (RH), II (PLK), and III (CTG) was selected and grown in tissue culture in either pH neutral conditions, conducive to tachyzoite growth or alkaline conditions, inducing bradyzoite differentiation. B) Multi-dimensional scaling (MDS) plot based on pairwise comparisons for each of the six experimental conditions. This is calculated as the root mean square deviation for the 500 most differentially expressed genes between any two conditions. The distance between any two points represents the average difference in expression levels (RPKM) of the most dissimilar genes, relative to differences observed between other conditions. In effect, the MDS plot provides an overview of the total amount of variation between samples. The axes show arbitrary distances. Experimental conditions include parasite strain (red  =  RH, green  =  PLK, blue  =  CTG) and by life cycle stage (X =  tachyzoite, O =  bradyzoite). Distances calculated using the 'plotMDS' function in the 'limma' Bioconductor package [30]. C) Boxplot represents absolute value of expression level difference between conditions. Interquartile regions are in gold, median differences are plotted as a solid black line. The root mean square deviation for each comparison is represented as a blue cross. From left to right, the first three groups are the intrastrain comparisons, tachyzoite vs. bradyzoite. The next three groups are interstrain comparisons between tachyzoite (unstressed) groups. The final three are are interstrain comparisons between bradyzoite (alkaline-stressed) groups).

We analyzed steady state mRNA levels from three different strains of *T. gondii*, RH (Type I, the most common laboratory strain), PLK (Type II, a clone of ME49 the genome reference strain), and CTG (Type III). We grew each strain under both “normal” tachyzoite tissue culture (pH 7, 5% CO_2_) conditions as well as bradyzoite-cyst inducing stress conditions (pH 8, low CO_2_), resulting in six total groups. Each condition was sampled in three biological replicates at a single time point. We had expected that this simple experimental design would yield groups of genes linked to the stress response, but the most striking observation is that the genes' steady state RNA levels vary much more between strains than between different stages of their life cycles. Figure 1B illustrates multi-dimensional scaling (MDS) of samples by condition, based on the top five hundred most divergent genes between each condition. The conditions cluster first by strain and then by life cycle stage. While tachyzoites and bradyzoites from different strains have many functional similarities, these results reinforce the divergence of gene expression of these parasites despite relatively modest differences in genome sequence.

The MDS plot only examines those genes with the most divergent expression levels. We also examined differences between conditions for all genes. First, we computed the differences in gene expression between tachyzoites and bradyzoites for each strain (three comparisons). We then examined differences between strains for both tachyzoites and bradyzoites (six more comparisons). The results are illustrated in Figure 1C. For all nine comparisons, the median and the interquartile ranges change very little and remain relatively close to zero. The mRNA levels of the majority of genes are not widely different between the strains and life cycle stage. To measure the total amount of change between compared groups, we calculate the root mean square difference (RMSD) for each of the nine comparisons. The RMSD values, plotted as blue crosses on Figure 1C, are much greater for the inter-strain comparisons than for the alkaline stressed/CO_2_ starved parasites of the same strain. This supports the observations shown in Figure 1B and further suggests that variation between the groups is driven by relatively small subsets of genes.

Comparisons between transcriptomes of tachyzoites and in vitro bradyzoites of the same strain are consistent with what is known about the propensity of each of these strains to differentiate. Type III (CTG) parasites readily switch from tachyzoite to bradyzoite and showed the greatest change in mRNA steady state levels, whereas Type I (RH) parasites fail to differentiate under these stress conditions and likewise showed almost no difference in mRNA between tachyzoite and bradyzoite conditions. Type II (PLK) parasites are intermediate to types I and III both in terms of differentiation competence as well as overall changes in its transcriptome. This is an important proof of principle that this type of global transcriptomic analysis does correlate with actual biological states of the parasite.

### Strain specific expression differences

**Figure 2:**
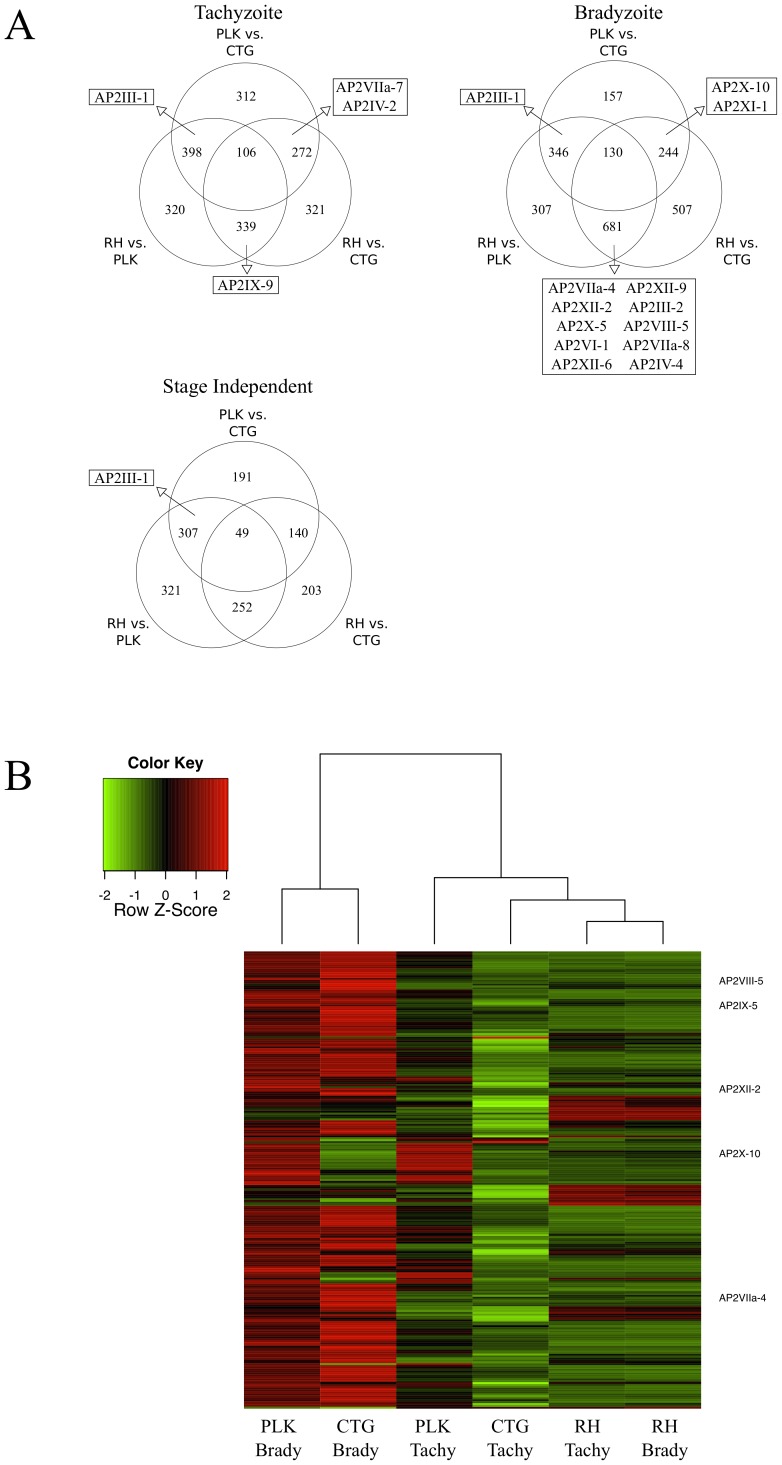
Hundreds of genes differentially expressed between strains including potential AP2 regulators. A) Using edgeR, we identify genes that are differentially expressed between strains (FDR <10%), generating three gene lists for tachyzoite condition and three for the bradyzoite condition. We generated Venn diagrams showing the overlap between the lists in the tachyzoite condition and bradyzoite condition. We also compared tachyzoite and bradyzoite gene lists to each other. Venn diagrams shows comparison of genes that appear in both the tachyzoite and the bradyzoite lists and therefore are “stage independent”. AP2 containing genes appearing on more than one list (any intersection) are indicated. The complete set of genes that are differentially expressed are listed in Table S2 and RPKM values for all replicates are listed in Table S1. B) Heat map of genes differentially expressed following CTG bradyzoite differentiation in all six conditions. Red indicates up regulation compared to that gene's expression level under other conditions, whereas green indicates down regulation. Conditions (columns) are clustered based on similarity of expression levels. The five AP2 genes that are differentially expressed are indicated on the right. Heat map was generated using the 'heatmap.2' function in the 'gplots' package for R. Hierarchical clustering of both the rows (genes) and columns (conditions) computed by the 'hclust' function in the R 'stats' package. Based on mean of replicate RPKM values.

Globally, we see a much greater difference between the transcriptomes of different strains than we do between stressed and unstressed parasites of the same strain (Figures 1B and 1C). To identify which genes are expressed differently between strains, we used edgeR. We compared each strain against the other two under both tachyzoite (unstressed) and bradyzoite (stressed) conditions (FDR 10%). This created six lists of differentially expressed genes that are differentially expressed between strains in either tachyzoites or bradyzoites as well those genes that were differentially expressed in both stages (Figure 2A). The number in the center of the diagram represents the number of genes differentially expressed between all three strains. A full gene list is in Table S2. Genes in the outer intersections differed in only one strain and those not falling into any intersection are cases where the expression level is different between two strains, but the strain's expression level was not significantly different. It is important to note that the number differentially expressed genes between any two strains is always equal to or larger than number of genes affected by differentiation.

If differential gene expression has indeed played an important in the evolution of *T. gondii* lineages, then regulators of transcription are likely candidates as drivers of evolution. Therefore, we examined whether AP2 genes are differentially expressed between strains. mRNA of seven AP2 differed in tachyzoites and mRNA levels of twenty- four AP2 differed among the bradyzoite transcriptomes. Five AP2 genes are differentially expressed between strains regardless of developmental stage. The AP2 genes that are differentially expressed in at least two comparisons are shown in Figure 2A and a full list is available in the Supplementary tables.

Steady state mRNA levels of AP2III-1 are significantly higher in PLK parasites than in either RH or CTG. It is a defining feature of the PLK strain regardless of developmental stage. Compared to PLK and CTG, expression of AP2VIIa-7 is lower in CTG tachyzoites, while expression of AP2IV-2 is higher. In CTG bradyzoites, expression of AP2X-10 and AP2XI-1 is lower than the other strains. RH tachyzoites express AP2IX-9 mRNA at significantly higher levels than either of the other strains examined. This is consistent with this transcription factors' reported role as a repressor of bradyzoite commitment [15], but differs from the pattern of expression initially reported. This difference may reflect that in our study parasites were predominantly the more stressed extracellular forms, in contrast to the intracellular parasites characterized previously. One can hypothesize that high levels of AP2IX-9 contributes to the parasite's ability to withstand stress and its inability to differentiate. Ten AP2 genes are differentially expressed in RH under stress conditions. This may reflect that RH does not differentiate into bradyzoites.

Expression of a number of AP2 genes in our study differs from previous reports that have compared AP2 expression in different strains. Some of these differences may be due to technical reasons such as inaccurate gene models that resulted in incorrect hybridization probes. Other differences are likely due to differences in experimental conditions. As proposed in earlier studies [16] and confirmed by GSEA analysis [22], extracellular tachyzoites represent an intermediate cell cycle arrested state with upregulation of stress-response genes that may be amongst the first that are induced during bradyzoite formation.

If experimental evidence supports the hypothesized role of AP2 genes as bona fide transcription factors, then the genes which they regulate are likely critical in determining phenotypic differences between strains. Differences in expression of AP2 in extracellular vs intracellular parasites may reflect expression differences seen in different biological states.

### Genes and genes pathways associated with *in vitro* stage differentiation

Using the edgeR package from the Bioconductor project, we identified 241 genes that were differentially expressed between CTG tachyzoites and bradyzoites with a false discovery rate (FDR) of 10%. Figure 2B is a heatmap of these genes across all six groups. Both PLK and CTG showed differential expression of these genes, whereas RH parasites are largely insensitive to alkaline stress treatment. Notably, only 33 of the 241 genes (13.7%) are down-regulated in the CTG bradyzoites.

Of the 241 genes related to CTG differentiation identified, there are five genes that are predicted to have an AP2 DNA binding domain [10]. AP2VIIa-4, AP2VIII-5, AP2IX-5, and AP2XII-2 are all up-regulated after 72 hours of *in vitro* bradyzoite conditions, while AP2X-10 is down-regulated. Members of this family have been shown to regulate transcription in malarial parasites, including differentiation to gametocytes [11] [12] [13]. One possibility is that these AP2 transcription factors are important for long-term maintenance of tissue cysts, either promoting the expression of bradyzoite-specific factors or repressing tachyzoite differentiation. Alternatively, these may be only transiently expressed during differentiation, and their temporal expression may reflect a cascade of events that occurs during developmental transitions. Some of the AP2 previously reported to be induced in bradyzoites [22] [23] were down-regulated in stressed CTG, supporting this hypothesis (Table S3). The expression of AP2VIIa-4 and AP2XII-2 are both linked to the cell cycle [8]. Given the associated between cell cycle and differentiation [24], it is not surprising that putative cell cycle transcription factors are differentially expressed following differentiation.

Examining individual genes can be a useful method of analyzing expression data. Understanding how many genes are affected and how much their expression changes provides an important global overview of the transcriptome under different conditions. This type of analysis also provides an unbiased way testing whether particular genes of interest are differentially expressed. In this case, we identified five potential transcription factors belonging to the family AP2. This association is intriguing and suggests a series of genetics experiments that could test if these factors contribute to *in vitro* cyst formation and maintenance.

There are, however, limits to a gene-by-gene analysis. For instance, we were unable to detect differentially expressed genes in either of the RH or PLK strains. RH is a type I parasite and therefore known to be resistant to stress-induced differentiation, but the PLK strain belongs to the type II lineage and is competent to differentiate. Although many of the genes differentially expressed in CTG had a similar pattern of expression PLK, the difference in expression was too low or the variance between replicates too high to achieve statistical significance. The number of required replicates to conclusively discriminate consistent differences is often prohibitively high. In addition, since analyses of this kind involve testing several thousand hypotheses, managing false positive inferences very often makes it impossible to distinguish signal from noise.

**Figure 3:**
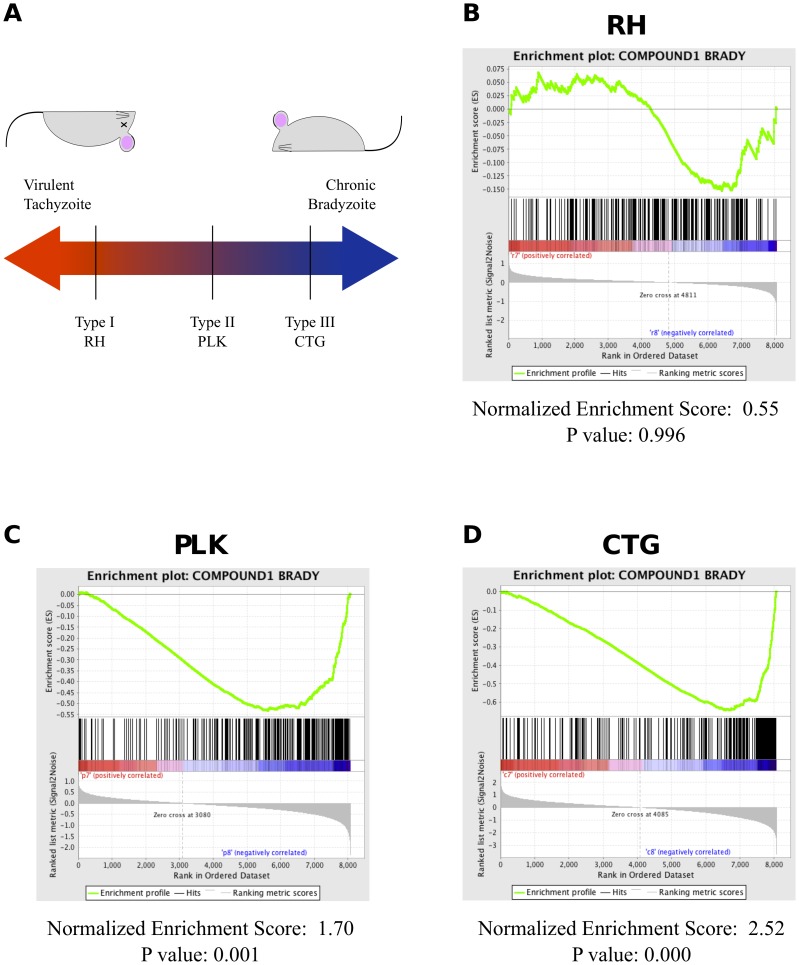
GSEA detects bradyzoite-induced genes in PLK and CTG, but not RH parasites. (A) A schematic of strain virulence of the strains used as a function of ability to differentiate into bradyzoites. (B) GSEA enrichment plot for RH parasites under differentiation conditions compared to compound1 induced genes. Position of black bars indicate ranking of compound 1 genes relative to all other genes. Green line represents strength of enrichment under bradyzoite conditions (right) or tachyzoite conditions (left). (C) Enrichment plot for PLK parasites under differentiation conditions compared to compound 1 induced genes. (D) Enrichment plot for CTG parasites under differentiation conditions compared to compound 1 induced genes.

Using GSEA, we compared the expression data to a set of genes shown to be differentially expressed after Compound 1 induced differentiation [22]. As Figure 3 shows, we are able to characterize both CTG and PLK, but not RH, as strongly enriched for bradyzoite genes. CTG has quantifiably more enrichment with a normalized enrichment score (NES) of 2.5 (p = 0.000) than PLK with an NES of 1.7 (p = 0.001). This is consistent with a continuum of differentiation competence with the type I lineage very resistant to bradyzoite development, type III differentiating readily, and type II parasites falling somewhere in the middle. Interestingly, the enrichment plot for RH (fig. 3B) is actually shaped like those of PLK, CTG (figs. 3C & 3D), even though the enrichment in RH is not statistically significant. This is consistent with data from other groups that indicates that RH is able to induce many of the stress-associated genes linked with bradyzoite differentiation, but is not able to complete the developmental transition.

### Strain specific metabolic differences

Presently, sequence homology is the primary method of predicting gene function. This poses a difficult problem for divergent eukaryotes like *T. gondii*, where approximately half of protein coding genes remain unannotated. The Kyoto Encyclopedia of Genes and Geneomes (KEGG) curates a collection of molecular pathways [17]. To begin parsing out the biological meaning of many differences in transcriptomes between the strains we used Gene Set Enrichment Analysis (GSEA) software [7] a tool that examines expression data holistically using “functionally related gene sets”, rather than testing genes individually. 33 KEGG pathways have been identified in *Toxoplasma* and are an appropriate size for use with GSEA. Tables 1 and 2 summarize the significant results for the interstrain comparisons in tachyzoites and bradyzoites, respectively.

**Table 1:** Differential expression of KEGG pathways between strains in the tachyzoite stage.

RH vs. PLK		RH vs. CTG		PLK vs. CTG	
RH	PLK	RH	CTG	PLK	CTG
			TGO03010: Ribosome		TGO03010: Ribosome
			TGO03008: Ribosome Biogenesis in Eukaryote		TGO03013: RNA Transport
			TGO03040: Spliceosome		TGO03008: Ribosome Biogenesis in Eukaryote
			TGO03013: RNA Transport		TGO03020: RNA Polymerase

**Table 2:** Differential expression of KEGG pathways between strains in treated with alkaline stress bradyzoite induction conditions.

RH vs. PLK		RH vs. CTG		PLK vs. CTG	
RH	PLK	RH	CTG	PLK	CTG
	TGO03030: DNA Replication		TGO03010: Ribosome		TGO03010: Ribosome
			TGO03008: Ribosome Biogenesis in Eukaryotes		TGO03008: Ribosome Biogenesis in Eukaryotes
			TGO03030: DNA Replication		TGO03013: RNA Transport
			TGO00240: Pyrimidine Metabolism		TGO00240: Pyrimidine Metabolism
					TGO03020: RNA Polymerase
					TGO03050: Proteasome

We find very few KEGG pathways enriched in any of the examined conditions. The most prominent result from this analysis is the much higher expression level of ribosomal proteins in CTG. In the absence of other stress, depletion of ribosomal protein RPS13 has been shown to arrest the cell cycle and induce BAG1 expression, but the parasites do not form a mature cyst wall, suggesting a state of partial differentiation [18]. It is not immediately clear why CTG expresses more ribosome components, but translational regulation has been linked to both the stress response and bradyzoite differentiation in *T. gondii* [19].

Thorough annotation of the *Toxoplasma* gene pathways will likely yield more insights into the biological processes underlying the divergence of the strains. The Liverpool Library of Apicomplexan Metabolic Pathways (LAMP) is one such thorough annotation and a promising starting point to address strain-specific differences [20]. However, by GSEA, we were unable to detect enrichment of any LAMP pathway (data not shown). The pathways we tested may not be expressed differently between strains, or the small size of the pathways may contribute to false negative results.

It is possible that despite the large differences in expression we observed between strains, there are not significant changes in the overall biological processes of the parasite between tachyzoites and bradyzoites. Recently Behnke showed that by principal components analysis, the transcriptomes of tachyzoites and bradyzoites group closely when compared to the transcriptomes of merozoite sexual stages harvested from cat intestines or the transcriptome of oocysts [21]. These findings speak to the importance of pathway analysis and global analysis over more traditional gene-by-gene testing.

### Differentiation influences the cell cycle

By examining DNA content, it has been shown that cell cycle arrest accompanies bradyzoite differentiation [24]. More recent work has identified two large sets of cell cycle regulated genes, with one set corresponding to the G_1_ phase and the other related to genes involved in S-phase and mitosis (S/M) [8]. To test how these cell cycle genes are affected by differentiation stress, we plotted the difference of each gene's expression level between tachyzoite conditions and bradyzoite-inducing stress conditions for each of the three parasite strains.

**Figure 4:**
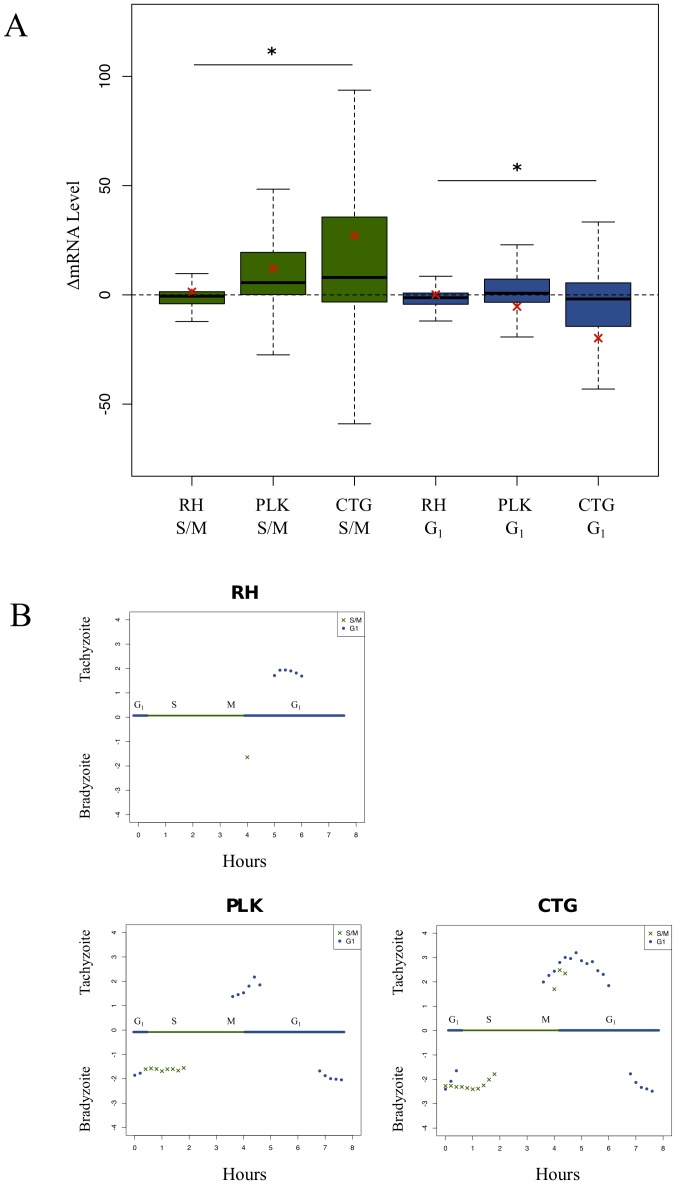
Expression of cell-cycle genes altered during differentiation. A) Boxplot represents differences in expression levels of cell cycle-dependent genes following differentiation conditions. Green boxes represent changes in S/M gene expression, blue boxes represent changes in G_1_ gene expression. A positive difference indicates up regulation of the gene in tachyzoite conditions, while a negative difference in expression values indicates greater expression in the alkaline stress induced bradyzoites. The black bar indicates median value; the red cross indicates mean value. Significance tested by one-way ANOVA. A star (*) indicates: p<0.001. B) Cell cycle genes are annotated as either G_1_ or S/M [8]. We then sorted genes into groups based on time of peak expression. Each of these gene sets was tested by GSEA [26]. Gene sets with significant (FWER-p value <0.05) normalized enrichment scores (NES) are plotted. Positive scores indicate association with the unstressed (tachyzoite) condition, negative scores indicate association with the stress (bradyzoite) condition. Blue and green bar across middle of plots represent an eight hour RH tachyzoite cell cycle. Counter-clockwise from the top, the plots show cell cycle gene sets influenced following RH differentiation, cell cycle gene sets influenced following PLK differentiation, cell cycle gene sets influenced following CTG differentiation. Note that time of expression is based on the RH cell cycle as defined, which is shorter than that of either PLK or CTG.

In keeping with the existing model, Figure 4A shows that S/M genes are more highly expressed in bradyzoite populations while G_1_ genes are more closely associated with tachyzoites. In bradyzoite differentiated parasites, there is an up regulation of S/M associated genes, while tachyzoites have higher steady state expression levels of G_1_-linked genes. The mean difference of each group (red cross) also illustrates these relationships. Further, there is again a clear difference in how each strain is affected by stress (significant difference of means by ANOVA). The cell cycle genes of RH are relatively unaffected by differentiation stress, while PLK experiences significant changes in expression of these genes and CTG more so. These data underscore the fundamental link between cell cycle regulation and life cycle advancement.

In the present model, the “switch” between tachyzoite and bradyzoite occurs in the late S phase [25]. This fits with the observed changes in gene expression, since not every cell cycle regulated gene is influenced the same way following differentiation stress. In Figure 4A, we show that the mean expression values change proportionate to each strain's ability to differentiate, but so does the variance. Not only are many of these gene unaffected by stress, but many of their expression values change in the “wrong” direction, with S/M annotated genes being up regulated in tachyzoites and G_1_ genes up regulated in bradyzoites.

To understand more precisely how bradyzoite development affects expression of cell cycle genes, we again employed GSEA. This time, we assigned cell-cycle genes into gene sets based on their peak time of expression [26]. In figure 4B, PLK and CTG bradyzoites show clear enrichment of multiple S phase gene sets, while tachyzoites are enriched for G_1_ gene sets with peak expression between hours four and six. Interestingly, many later G_1_ gene sets are actually associated with bradyzoites. In this analysis, we also find that the RH cell cycle is affected by the stress conditions, but to a much lesser extent than PLK or CTG.

The cell cycle transcriptome is temporally linked with differentiation stress. Using GSEA, we are able to identify specific cell cycle genes, grouped by time of peak expression, that are linked with bradyzoites or tachyzoites. Once again, the continuum of virulence (Figure 3) is observable. Both the number of the gene sets enriched and the magnitude of the enrichment is great in CTG than PLK. What is most interesting is that the cell cycle of RH parasites does appear to affected by differentiation stress, albeit to a much lesser extent than either of the other two strains. This was the only indication that RH parasites reacted to the alkaline stress conditions and probably speaks to the fact that disruption of the cell cycle precedes the bradyzoite developmental switch.

### Conclusions

In summary our integrative analysis of RNA-seq points to strain specific differences in gene expression that are more prominent than changes in gene expression associated with exposure to alkaline stress. The comparison of strains with different virulence phenotypes and capacity to differentiation using various tools has enabled us to identify pathways and genes that may be conserved between strains as well as those that regulate strain-specific traits.

## Materials and Methods

This work did not involve human subject or animal research. All procedures were approved by the appropriate biosafety committees at the Albert Einstein College of Medicine.

### Parasite culture

Human foreskin fibroblasts (HFF) cells grown in eight 150 mm tissue culture plates were infected with RH (type I), PLK (type II), and CTG (type III) strain in regular medium (pH 7 DMEM with 10% fetal bovine serum, incubated in 5% CO_2_). The uninvaded RH free parasites were removed 2 hours after inoculation while the CTG and PLK strain free parasites were removed 4 hours later by washing with PBS. Inoculation medium was and then replaced with regular medium (pH 7, 5% CO_2_) or differentiation medium (pH 8.1 DMEM with 5% fetal bovine serum, 10 mM HEPES, incubated in 0.5% CO_2_). Thethe RH infection duration was two days while CTG and PLK strain were three days. Tachyzoite preparations were a mix of freshly lysed extracellular parasites and mature vacuoles on the verge of lysis as were alkaline-shocked RH strain. Afterwards, cells were harvested, passed through 27G needle twice to lyse HFF cells and filtered through 3 µm pore polycarbonate membrane to remove HFF cells. Purified parasites were pelleted at 1000 xg for 20 minutes 4°C. Pellets were stored in TRIzol Reagent (Invitrogen) in −80°C.

### Library preparation and sequencing

RNA was extracted using Invitrogen TRIzol Reagent (cat#15596018), followed by genomic DNA removal and cleaning using Qiagen RNase-Free DNase Set kit (cat#79254) and Qiagen Mini RNeasy kit (cat#74104). Agilent 2100 Bioanalyzer was used to assess the integrity of the RNA samples. Only RNA samples having RNA Integrity Number between 9–10 were used. Ambion MicroPoly(A)Purist Kit (cat#AM1919) was used for enrichment of transcripts. The SOLiD Total RNA-Seq Kit (cat#4445374) was used to construct template cDNA for RNA-Seq following the protocol recommended by Applied Biosystems. Briefly, mRNA was fragmented using chemical hydrolysis followed by ligation with strand specific adapters and reverse transcript to generate cDNA. The cDNA fragments, 150 to 250 bp in size, were subsequently isolated by electrophoresis in 6% Urea-TBE acrylamide gel. The isolated cDNA was amplified through 15 amplification cycles to produce the required number of templates for the SOLiD EZ Bead system, which was used to generate template bead library for the ligation base sequencing by the SOLiD4 instrument.

We aligned sequenced RNA fragments against release 6.1, ME49 strain of *Toxoplasma gondii* [27] using TopHat-1.2 [28]. We set minimum intron size at 30 bp and maximum at 1500 bp, encompassing *>*98% of predicted introns [27]. All other parameters were left at their default values. We assigned aligned reads to predicted gene models using BEDTools [29] and generated a table describing how many reads are aligned to each gene.

Using this “counts” table, we generated the unsupervised clustering or multi-dimensional scaling plot in figure 1A with the “plotMDS” function from the Limma package [30] and “predFC” from edgeR package [31]. Both packages are available through the Bioconductor project. Presented data is the mean of three RPKM normalized [32] replicates for each condition. All data generated in this study are accessible at the GEO database under accession number GSE60305. A summary of RPKM for each version 6.1 gene is listed in Table S1. Data have also been provided to the community database www.toxodb.org.

### Detection of differential gene expression

We used the edgeR software package to infer differential expression [31]. Briefly, each gene across all eighteen samples was fitted with a log-linear model, then the regression coefficients for each group (both strain and growth conditions) are compared. Inequality of regression coefficients strongly suggests differential expression. Differentially expressed genes are listed in Table S2.

### Gene Set Enrichment Analysis

GSEA is a pathway analysis tool supported by available through the Broad Institute (http://www.broadinstitute.org/gsea/index.jsp). Subramanian and colleagues describe the algorithm in detail [7]. We permuted by gene sets, not phenotypes and used the “Signal2Noise” ranking metric. Gene sets were based on KEGG pathway annotation [17], while gene sets related to cell cycle and differentiation were developed for implementation of GSEA for *T. gondii* [26].

## Supporting Information

Table S1 **RPKM normalized expression values for all genes, across all samples (TgME49_6.1).** RNA-seq transcriptome data expressed as RPKM for each of 3 biological replicates grown and harvested at pH 7 or pH 8. r = RH; p = PLK; c = CTG.
(XLS)

Table S2 **Membership table for genes differentially expressed in different pairwise comparisons.** The genes are as listed in Table S1, using the version 6.1 gene ID's obtained from www.toxodb.org used to initially map the reads. All genes that were found to be differentially expressed in the comparisons discussed in the text are marked with an 'x'. Cell cycle regulated genes (G1 and S/M) and AP2 genes as defined by Behnke [8] are also indicated. Definition of abbreviations: RH → RH – Tachy vs Brady; PLK → PLK – Tachy vs Brady; CTG → CTG – Tachy vs Brady; rpt → RH vs PLK (Tachyzoite); rct → RH vs CTG (Tachyzoite); pct → PLK vs CTG (Tachyzoite); rpb → RH vs PLK (Bradyzoite); rcb → RH vs CTG (Bradyzoite); pcb → PLK vs CTG (Bradyzoite); g1 → Annotated as G1 cell cycle gene; sm → Annotated as S/M cell cycle gene; ap2 → Predicted or confirmed AP2 DNA-binding domain.
(XLS)

Table S3 **RPKM expression values for annotated AP2 genes.** Product names are those conferred by a community annotation group as available on www.toxodb.org. Gene ID's are version 6.1. Developmental assignments are per Behnke [8] and Walker Mol Micro [23]. Mean RPKM for each strain and condition are shown: r7 (RH strain pH 7); r8 (RH strain pH 8); p7 (PLK strain pH 7); p8 (PLK strain pH 8); c7 (CTG strain pH 7); c7 (CTG strain pH 8);
(XLS)
